# Draft Genome Sequence of Lactobacillus kunkeei AR114 Isolated from Honey Bee Gut

**DOI:** 10.1128/genomeA.00144-15

**Published:** 2015-03-19

**Authors:** Davide Porcellato, Cyril Frantzen, Anbjørg Rangberg, Ozgun C. Umu, Christina Gabrielsen, Ingolf F. Nes, Gro V. Amdam, Dzung B. Diep

**Affiliations:** a Department of Chemistry, Biotechnology and Food Science, Norwegian University of Life Sciences, Ås, Norway; b Arizona State University, School of Life Sciences, Tempe, Arizona, USA

## Abstract

Lactobacillus kunkeei is a common inhabitant in honey bee gut, being present in several parts of the world. Here, we describe the draft genome of L. kunkeei AR114, an isolate from late foraging season in Norway.

## GENOME ANNOUNCEMENT

Lactobacillus kunkeei is a common species in the gut of honey bees and has been considered to have a great potential in paratransgenesis and as a gene/protein delivery vehicle in honey bees (1–4). We have recently observed that its presence is seasonal dependent. The bacterium is prevalent in the gut during foraging season but disappears during the Nordic winter season when the bees start receiving a defined mixture of sugars as food replacement (4). A collection (*n* = 40) of L. kunkeei isolates with great genetic variation in plasmid content have been isolated from the foregut of bees during the late foraging season in Norway (September to December in 2001) (2).

The genome of L. kunkeei AR114, isolated in the month of December, was sequenced from good-quality DNA isolated using a Genomic Tip kit (Qiagen, Germantown, MD, USA). DNA was sequenced on an Illumina MiSeq platform at the Department of Chemistry, Biotechnology and Food Science (Norwegian University of Life Sciences, Ås, Norway) and at the Norwegian Sequencing Center (University of Oslo, Oslo, Norway). A 300-bp paired-end library was constructed using a Nextera XT kit (Illumina, San Diego, CA, USA). Reads were quality filtered using Nesoni and *de novo* assembled using Ray assembler (5). Contigs with length below 1,000 bp and coverage below 5× were removed. Coding DNA sequences (CDS) were predicted and annotated using NCBI Prokaryotic Genome Annotation Pipeline (6).

The draft genome of L. kunkeei AR114 consists of 21 contigs for a total of 1,557,121 bp with a GC content of 36.9%. The largest contig is 358,782 bp. The total number of CDS was 1,297 and the number of RNAs was 70. One confirmed clustered regularly interspaced short palindromic repeats (CRISPR) array was identified using CRISPRfinder (7). This CRISPR array contains 28 spacers and has length of 1,883 bp.

The availability of this draft genome may contribute to better understanding of the geographical, biological, and ecological role of gut microbes in insects, and more specifically, L. kunkeei in honey bees, and also to help develop much needed genetic tools for studies in bees.

### Nucleotide sequence accession number.

This whole-genome shotgun project has been deposited at DDBJ/EMBL/GenBank under the accession number JPYX00000000. The version described in this paper is the first version.
